# γδ T cell costimulatory ligands in antitumor immunity

**DOI:** 10.37349/ei.2022.00038

**Published:** 2022-02-24

**Authors:** Joseph M. McGraw, Deborah A. Witherden

**Affiliations:** 1 Department of Biology, Calibr at The Scripps Research Institute, La Jolla, CA 92037, USA; 2 Department of Immunology and Microbiology, The Scripps Research Institute, La Jolla, CA 92037, USA

**Keywords:** γδ T cell, costimulation, natural killer group 2D, junctional adhesion molecule-like protein, CD100, lymphocyte function-associated antigen 1, CD316

## Abstract

Antitumor immunity relies on the ability of T cells to recognize and kill tumor targets. γδ T cells are a specialized subset of T cells that predominantly localizes to non-lymphoid tissue such as the skin, gut, and lung where they are actively involved in tumor immunosurveillance. γδ T cells respond to self-stress ligands that are increased on many tumor cells, and these interactions provide costimulatory signals that promote their activation and cytotoxicity. This review will cover costimulatory molecules that are known to be critical for the function of γδ T cells with a specific focus on mouse dendritic epidermal T cells (DETC). DETC are a prototypic tissue-resident γδ T cell population with known roles in antitumor immunity and are therefore useful for identifying mechanisms that may control activation of other γδ T cell subsets within non-lymphoid tissues. This review concludes with a brief discussion on how γδ T cell costimulatory molecules can be targeted for improved cancer immunotherapy.

## Introduction

γδ T cells are a unique lymphocyte population that has important roles in tissue homeostasis, infection, and cancer. Although γδ T cells are a minor population within lymphoid tissues (typically 1–5% of T cells in peripheral blood), they are present in larger numbers in both mouse and human epithelial tissues (10–100% of T cells) such as the skin and gut [1]. Within these tissues, γδ T cells detect signals of cellular stress that are increased following tissue damage or malignant transformation, and upon activation, they produce cytokines, chemokines, and growth factors that promote tissue repair and eliminate developing tumors [1]. The critical role of γδ T cells in antitumor immunity is highlighted by the fact that mice lacking these cells T cell receptor delta-deficient (*Tcrd*^*−/−*^) are more susceptible to tumorigenesis in a variety of preclinical mouse tumor models [2–4]. Although some protumor roles of γδ T cells, often associated with interleukin-17 (IL-17) production, have been described in both mouse models and human disease, γδ T cells are generally associated with potent antitumor immunity [5]. For example, the presence of γδ T cells within solid tumors has been identified as a strong prognostic factor for overall survival in patients in both meta-analyses [6] and recent, individual case studies of solid tumors such as Merkel cell carcinoma [7], breast cancer [8], and ovarian cancer [9]. Similar to their αβ T cell counterparts, the antitumor functions of γδ T cells include direct lysis of tumor targets and secretion of inflammatory cytokines such as interferon-γ (IFNγ) that promote broader antitumor immunity. However, γδ T cells recognize antigens independent of major histocompatibility complex (MHC) presentation and detect self-stress molecules associated with tissue damage and malignancy, which allow them to provide rapid, innate-like responses within non-lymphoid tissues [5]. This unique feature of γδ T cells is exemplified by the ability of both mouse and human γδ T cells to lyse a broad range of tumor targets *in vitro* [10]. The study of γδ T cells is complicated by the evolutionary divergence of T cell receptor (*TCR*) genes between mouse and human (Table 1) and the limited understanding of *bona fide* γδTCR antigens, which has recently been reviewed elsewhere [11]. Despite these challenges, the study of γδ T cells in mice has led to the identification of key molecules important for their maintenance of tissue homeostasis and regulation of antitumor immunity that may have relevance for the treatment of human disease. Here, activating receptor-ligand interactions important for the activity of γδ T cells within non-lymphoid tissues are reviewed, with a specific focus on mouse dendritic epidermal T cells (DETC), and their potential roles in antitumor immunity, concluding with a brief discussion on how this knowledge could be leveraged for improved cancer immunotherapy.

### DETC

DETC are a prototypic tissue-resident γδ T cell population in mouse skin that has critical roles in the maintenance of tissue homeostasis, wound healing, and tumor immunosurveillance [13]. Under homeostatic conditions in the mouse epidermis, DETC display a characteristic dendritic morphology that allows them to survey neighboring keratinocytes [14]. DETC express an invariant, canonical Vγ3^+^Vδ1^+^ TCR (Garman nomenclature) that recognizes an unidentified self-stress ligand [15]. Upon activation, DETC retract their dendrites and round up presumably to allow for migration towards signals of tissue insult where they secrete inflammatory cytokines such as tumor necrosis factor-α (TNF-α) and IFNγ as well as growth factors such as keratinocyte growth factor-1 (KGF-1) [16, 17]. The critical role of DETC in the maintenance of skin tissue homeostasis is highlighted by the fact that mice lacking DETC are more susceptible to carcinogen-induced skin tumor formation and exhibit delayed wound healing [2, 14].

**Figure 1.**
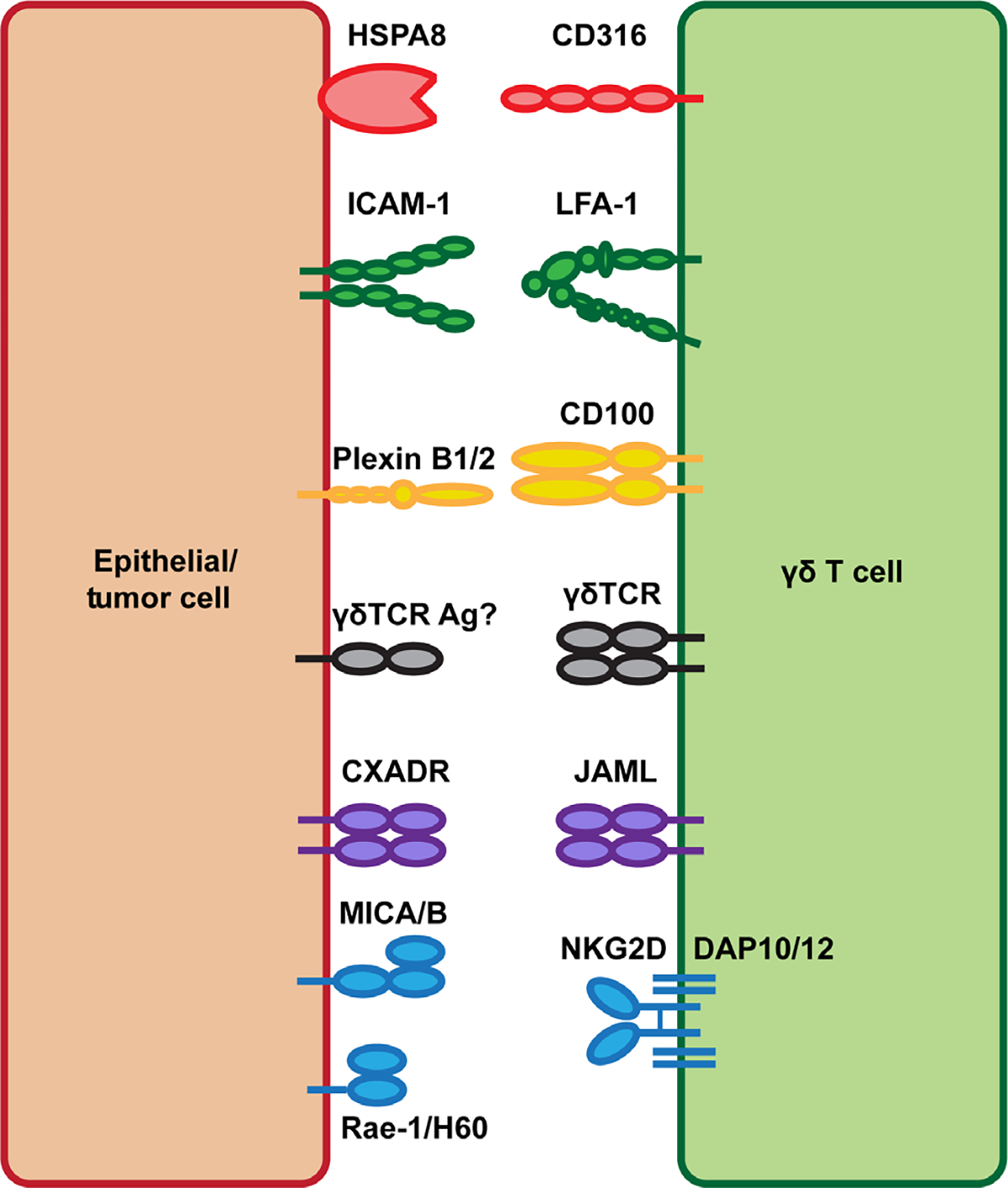
Tissue-resident γδ T cell costimulatory ligand interactions. Tissue-resident γδ T cells express the costimulatory molecules NKG2D, JAML, CD100, LFA-1, and CD316. Upon tissue damage or malignant transformation, epithelial cells upregulate expression of both γδTCR antigens and the costimulatory ligands Rae-1/H60 [MHC class I polypeptide-related sequence A and B (MICA/B) in human], CXADR, plexin B1/2, ICAM-1, and HSPA8 allowing for γδ T cell activation and their promotion of tissue homeostasis. HSPA8: heat shock protein family A member 8; ICAM-1: intercellular adhesion molecule-1; Ag: antigens; CXADR: coxsackie and adenovirus receptor; Rae-1: retinoic acid early inducible 1; H60: histocompatibility 60; DAP10/12: DNAX-activating protein 10/12

Like other γδ T cell populations in both mice and humans, DETC do not recognize antigens in the context of MHC antigen presentation and lack CD4, CD8, and CD28 co-receptors that are critical for the priming of αβ T cells [18]. As a result, the exact mechanisms required for DETC activation are less well-characterized. Investigators have sought to identify the key molecules important for the function of DETC. These studies have identified natural killer group 2D [NKG2D; encoded by killer cell lectin like receptor K1 (*Klrk1*)], junctional adhesion molecule-like protein (JAML), CD100 ([encoded by semaphorin 4D (*Sema4d*)], lymphocyte function-associated antigen 1 (LFA-1), and CD316 as activating co-receptors that are critical for DETC function (Figure 1). Each of these molecules has important role in antitumor immunity and is potential therapeutic target for improved cancer immunotherapy.

### NKG2D

NKG2D is a C-type lectin-like activating receptor expressed by natural killer (NK) cells, γδ T cells, and CD8 T cells that are critical for the recognition and elimination of damaged, infected, and cancerous cells. NKG2D interacts with the stress-induced ligands Rae-1, H60, and mouse unique long 16 (UL16)-binding protein-like transcript (Mult-1) in mouse and MICA/B and UL16-binding proteins 1–6 (ULBP1–6) in humans [19]. NKG2D associates with the adapter protein DAP10 in humans and either DAP10 or DAP12 in mice, which stabilizes the receptor complex and mediates intracellular signal transduction upon NKG2D ligand binding. Upon NKG2D activation, DAP10 recruits a p85 phosphoinositide 3-kinase (PI3K) and Vav-1 signaling complex, whereas DAP12 contains a canonical immunotyrosine-based activation motif (ITAM) that binds spleen tyrosine kinase (Syk) and Zeta-chain-associated protein kinase 70 (ZAP70) tyrosine kinases [20].

The function of NKG2D as an activating receptor important for antitumor cytotoxicity was initially discovered based on the observation that human Vδ1^+^ T cell clones recognize MICA/B expressed on a wide range of epithelial tumors including lung, breast, kidney, prostate, and colon cancers [21, 22]. MICA was found to bind to human NK cells and CD8, but not CD4, T cells and was identified as a ligand for NKG2D [21]. Shortly after this discovery of NKG2D-MICA/B interactions in humans, similar findings were reported in mouse tumor models. This seminal work showed that *Tcrd*^*−/−*^ mice were more susceptible to both growth of transplantable squamous cell carcinomas and formation of skin tumors induced by the chemical carcinogens demonstrating a key antitumor role for γδ T cells [2]. The authors additionally went on to show that this antitumor activity of γδ T cells was mediated by DETC, which killed tumor cells via NKG2D recognition of the mouse homologs of human MICA/B, Rae-1, and H60 [2].

Since these initial reports identifying NKG2D as a critical receptor for the antitumor activity of γδ T cells, many other groups have described important roles for NKG2D in γδ T cell biology with a number of studies focused specifically on NKG2D in the function of DETC in murine skin [23–27]. Together, these studies have highlighted NKG2D recognition of self-stress ligands such as Rae-1 and H60 as a key component of tissue-resident γδ T cell activation in response to a wide variety of tissue insults. Transgenic overexpression of Rae-1 in the epidermis of mice was found to result in DETC activation within a 5-day timeframe, as measured by DETC rounding, upregulation of CD69, and downregulation of γδTCR expression [23]. This result indicated that DETC are actively involved in tumor immunosurveillance and rapidly responds to stress signals in murine skin. In addition to roles in antitumor immunity, an important role for NKG2D in DETC function has been described in the homeostatic maintenance of skin tissue [24], atopy [25], cutaneous wounding [26], and contact hypersensitivity [27].

Unlike classical costimulatory molecules which only function in combination with TCR engagement, NKG2D has been proposed to activate DETC independent of TCR signals [28]. In the absence of TCR signals, NKG2D binding induces PI3K-dependent signaling that results in the DETC lysis of target cells *in vitro*. In contrast, IFNγ production is driven more potently by Syk/ZAP70 signaling downstream of TCR engagement [29]. Similar TCR-independent antitumor cytotoxicity through NKG2D by human Vγ9Vδ2 T cells has also been described. However, depending on the tumor target, Vγ9Vδ2 T cells can kill tumor targets via TCR engagement alone or require co-engagement of TCR and NKG2D [30]. Although the relative importance of TCR *versus* NKG2D signaling for γδ T cell activation remains controversial, it is clear that NKG2D is an important regulator of γδ T cell cytotoxicity against a broad range of both mouse and human tumor cell lines [31].

In addition to a critical role for DETC function, NKG2D has been shown to have a critical role in the antitumor activity of other mouse γδ T cell subsets. Mouse lymphoid Vγ2^+^ T cells possess antitumor activity against B16 melanoma cells *in vivo*, which was associated with inhibition of B16 cell growth, but not direct cytotoxicity, *in vitro*. Tumor suppressive activity of Vγ2^+^ T cells was dependent on both TCR and NKG2D signals, which regulated their production of IFNγ. This antitumor activity of Vγ2^+^ T cells can be further enhanced by treatment with the mTOR inhibitor rapamycin *in vitro*, which sensitized cells to IL-2 signals resulting in increased expression of both NKG2D and TNF-α and higher cytotoxicity. Vγ2^+^ T cells expanded *in vitro* with rapamycin had improved antitumor activity against B16 melanoma *in vivo* upon adoptive cell transfer (ACT) compared with cells expanded with TCR and CD28 signals alone [32].

### JAML

JAML (also known as *AMICA1* in humans) is a member of the junctional adhesion molecule family, a class of molecules that facilitate tight junction assembly, regulate leukocyte-endothelial interactions, and have diverse roles in development, angiogenesis, inflammation, and cancer [33, 34]. JAML binds to the CXADR, a cell adhesion molecule expressed by non-hematopoietic cells including epithelial cells within the skin and gut tissue [35–37]. JAML-CXADR interactions have been identified as a novel costimulatory mechanism for the activation of DETC. Upon binding to CXADR, JAML induces a PI3K signaling cascade, which promotes cytokine production and proliferation by DETC. CXADR is expressed at low levels in mouse skin under homeostatic conditions, but following cutaneous wounding, keratinocytes increase expression of CXADR allowing for increased JAML costimulation of DETC and their subsequent production of soluble factors such as KGF-1, which promote the wound repair process [37].

Based on this initial characterization of JAML-CXADR-mediated costimulation of tissue-resident γδ T cells and the known roles of γδ T cells in antitumor immunity, the importance of JAML-CXADR interactions for γδ T cell responses within the tumor microenvironment (TME) was investigated. Mice lacking JAML protein expression (*Jaml*^*−/−*^) were more susceptible to both B16F10 melanoma tumor formation and growth, which was associated with decreased activity of γδ T cells. During the early stages of tumor growth, *Jaml*^*−/−*^ mice had fewer numbers of IFNγ-producing γδ tumor-infiltrating lymphocytes (TIL) demonstrating that the γδ T cell response to B16 tumor growth is mediated, at least in part, by JAML-CXADR interactions. Importantly, this γδ TIL response consisted of Vγ1.1^+^, Vγ2^+^, and Vγ1.1^−^Vγ2^−^ infiltrating subsets, but not DETC, demonstrating a broader role for JAML-CXADR interactions in γδ T cell biology. Unlike DETC, which constitutively express JAML, naive lymphoid γδ T cells did not express high levels of JAML. However, lymphoid γδ T cell subsets upregulated JAML upon *ex vivo* stimulation and within the TME after which JAML engagement further enhanced activation and cytokine production. Therefore, JAML is also an important costimulatory ligand for lymphoid γδ T cell subsets after initial T cell priming [38].

CXADR expression is dysregulated in many human cancers [39]. For example, loss of CXADR expression is associated with increased epithelial-mesenchymal transition in breast cancer [40] and increased gastric cancer metastasis [41]. A similar association with decreased CXADR expression and increased malignancy was also observed in both mouse and human melanoma where CXADR expression was higher in benign lesions and during early stages of tumor growth but was decreased upon tumor growth and progression [38]. Furthermore, both mouse and human melanocytes express very low levels of CXADR suggesting that CXADR expression is linked to melanoma tumorigenesis [38]. During the early stages of the disease, this pattern of CXADR expression mirrors findings in cutaneous wound healing where expression is low under homeostatic conditions but is increased following tissue damage or malignant transformation. Together, these results suggest that CXADR is an important stress ligand that functions as a signal to tissue-resident γδ T cells to mediate both tissue repair and antitumor immunity.

In contrast to impaired antitumor immunity in the absence of JAML, treatment of tumor-bearing wild type (WT) mice with an anti-JAML agonistic antibody [37] significantly limited tumor growth and extended median survival. Furthermore, the anti-JAML treatment improved the efficacy of programmed cell death 1 (PD-1) blockade when used in combination. This effect of anti-JAML treatment was associated with improved markers of both CD8 and γδ TIL immunity in WT mice. Critically, in both *Tcrd*^*−/−*^ mice and mice treated with CD8 T cell depleting antibodies, the antitumor effect of anti-JAML treatment was not observed [38]. These results point to a key role of JAML-mediated γδ T cells responses in antitumor immunity and the necessary cooperation between CD8 and γδ T cells, which involves JAML-CXADR interactions.

### CD100

CD100 (also known as SEMA4D) is a group IV semaphorin expressed by T cells, B cells, platelets, NK cells, and monocytes and binds with low affinity to CD72 on other immune cells and with high affinity to plexin B1/2. Semaphorins are a large family of molecules that interact with plexins to regulate cytoskeleton dynamics and were initially discovered to have critical roles in neuronal development [42]. Studies of mice lacking CD100 (*Cd100*^*−/−*^) have since demonstrated key roles for CD100 in the regulation of both humoral and cellular immunity [43]. These studies have shown that CD100-CD72 interactions are critical for both T cell priming by antigen-presenting cells (APCs) and B cell proliferation and antibody production [44, 45]. CD100 can function as both a membrane-bound protein on the surface of immune cells and as a soluble molecule either following proteolytic cleavage by matrix metalloproteinases (MMPs) or cell-intrinsic shedding [46, 47]. In B cells, binding of either membrane-bound or soluble CD100 to CD72 displaces Src homology 2 domain-containing protein tyrosine phosphatase 1 (SHP-1) from its intracellular immunoreceptor tyrosine-based inhibitory motif (ITIM) domain allowing for increased activation [45]. On the other hand, CD100 binding to plexin B1 on epithelial and endothelial cells results in the recruitment of small GTPases, which facilitate changes in cytoskeleton dynamics [48].

Plexin B2 has been identified as a novel ligand for CD100, which is expressed by skin epithelial cells and regulates DETC function in mice [49]. Similar to the findings on JAML-CXADR regulation of DETC function, it was found that plexin B2 is expressed by keratinocytes under homeostatic conditions, is further upregulated upon cutaneous wounding, and interacts with CD100 on DETC to promote their wound healing activity. Upon binding to plexin B2, CD100 induces extracellular signal-related kinase (ERK) and cofilin signaling resulting in DETC rounding and increased integrin expression [49]. Additionally, CD100-plexin B2 regulation of γδ T cell-mediated tissue repair is critical in the dextran sodium sulfate (DSS) mouse model of colitis. In this model, activation of γδ intraepithelial lymphocytes (IEL) in the intestine by CD100-plexin B2 binding resulted in the production of KGF-1, which was required to limit the severity of colitis [50]. In addition to pro-wound healing functions in the skin and gut, CD100-plexin B2 interactions have been shown to be involved in psoriasis in both patients and mouse models [51]. Importantly, in the mouse model of psoriasis induced by treatment with the toll-like receptor 7 (TLR7) agonist imiquimod (IMQ), knockdown of plexin B2 in mouse keratinocytes *in vivo* limited inflammation and epidermal infiltration by dermal γδ T cells, which are known to be a critical source of IL-17 and drive inflammation in this model [51]. This result, together with the colitis model data, suggests that CD100-plexin B2 interactions may be more generally important for the regulation of γδ T cell function in response to tissue damage and inflammation.

Although a role for CD100 in γδ T cell antitumor immunity has not been described, CD100 does play an important role within the TME. In mouse tumor models, both genetic deletion (*Cd100*^*−/−*^) and antibody-mediated blockade of CD100 limit tumor growth highlighting a pro-tumorigenic role for CD100 signaling [52]. In humans, high CD100 expression has been associated with worse patient outcomes in colorectal [53], ovarian [54], sarcoma [55], and cervical [56] cancers. Because both tumor cells and immune cells can express CD100 and CD100 ligands, the signaling pathways involved in their promotion of tumor growth are complex. CD100 is highly expressed by tumor-associated macrophage (TAM) and interactions with plexin B1 within the TME promote tumor angiogenesis [57]. Tumor cells themselves can also secrete CD100, which polarizes myeloid cells towards an immunosuppressive myeloid-derived suppressor cell (MDSC) phenotype [58]. This promotion of MDSC is limited by treatment with a blocking anti-CD100 antibody, which improves CD8 T cell antitumor activity and synergizes with immune checkpoint blockade (ICB) in mouse tumor models [59].

Although most studies have focused on the roles of CD100 in myeloid cell and B cell function, CD100-CD72 interactions are also critical for CD8 T cell activation and proliferation. CD8 T cells upregulate both CD100 and CD72 upon activation after which CD100-CD72 interaction drive further CD8 T cell activation. In the context of cancer, a recent study of patients with non-small cell lung cancer found that CD8 T cells isolated from bronchoalveolar lavage fluid were activated by CD100 stimulation *ex vivo* and that decreased shedding of membrane-bound CD100 from T cells and the resulting decreased levels of circulating soluble CD100 may impair CD8 T cell function *in vivo* [60]. Therefore, CD100 interactions may be important for antitumor T cell responses, and the extent to which CD100 interactions regulate CD8 or γδ T cell function within the TME deserves further investigation. Given the data on the critical role of CD100 in DETC function, it is possible that plexin B2 may function as a tumor stress signal that is important for γδ T cell tumor immunosurveillance and promotion of antitumor immunity. Conversely, the clear antitumor effects of anti-CD100 blocking antibodies could involve inhibition of tumor-promoting IL-17^+^ γδ T cell function [61]. Parsing apart these roles for CD100 on myeloid cell and T cell function within the TME will help inform if CD100 is a viable therapeutic target for the treatment of human cancers.

### LFA-1

LFA-1 is an integrin heterodimer composed of CD11a and CD18 and has important roles in regulating T cell activation and migration. LFA-1 binds ICAM-1 allowing for prolonged T cell-APC interactions, increased tissue migration, and contact with target cells for cytolytic activity [62, 63]. LFA-1 binding also lowers the threshold required for T cell activation by signaling through cytohesin-1, which results in mitogen-activated protein kinase (MAPK) pathway activation, and through Jun activation domain-binding protein 1 (JAB-1), which promotes c-Jun phosphorylation. Together with TCR and CD28 signaling, these LFA-1 driven events promote cell proliferation and IL-2 production [64]. Although most work on LFA-1 has focused on its role in the regulation of αβ T cell responses, LFA-1 has long been known to be important for γδ T cell biology. LFA-1 has been shown to be important for γδ T cell cytotoxic activity against human melanoma [65], ovarian [66], lymphoma [67], myeloma [68], pancreatic [69], and lung cancer cell lines [70]. In this context of tumor cell killing, LFA-1 is critical for adhesion and immune synapse formation with target cells, which enhances γδ T cell cytotoxicity [70]. Although direct cytotoxicity was not measured, LFA-1 has also been shown to be critical for the adhesion of human Vδ1^+^ γδ T cells to esophageal squamous cell carcinoma cells [71]. Separately, a study using *Icam1*^*−/−*^ mice also identified a key role for LFA-1-ICAM-1 interactions in the migration and pro-wound healing function of tissue-infiltrating γδ T cells upon damage to the cornea epithelium [72].

In recent work, a role for LFA-1-ICAM-1 interactions in the function of DETC in response to skin wounding has been identified [73]. Using an unbiased ribonucleic acid (RNA) sequencing-based approach, ICAM-1 was identified as a keratinocyte stress ligand that was significantly upregulated in both mouse and human skin upon wounding. DETC were found to constitutively expresses LFA-1 and could be co-stimulated *in vitro* with either ICAM-1-fragment crystallizable (Fc) protein or agonist anti-LFA-1 antibodies. Importantly, *ex vivo* wound closure, a measure of DETC-mediated wound healing function independent of additional infiltrating immune cells, was impaired in skin tissue isolated from *Icam1*^*−/−*^ mice [73]. These findings are consistent with prior studies that reported delayed wound closure in both skin and gut tissue in *Icam1*^*−/−*^ mice. Upon wounding of both skin and gut tissue, ICAM-1 expression on epithelial cells was shown to be important for cell proliferation and infiltration of polymorphonuclear cells, which supported wound healing [74, 75]. Together, these results suggest that ICAM-1 on epithelial cells acts as a tissue stress ligand in a manner analogous to CXADR and plexin B2 allowing for tissue-resident γδ T cell activation and their maintenance of tissue homeostasis. Although LFA-1 is clearly important for γδ T cell lysis of tumor cells [65–70], additional characterization of LFA-1-ICAM-1 interactions in the regulation of γδ T cell migration, retention, and function within the TME would be of value. Given that LFA-1 and JAML function similarly as both adhesion and costimulatory molecules and studies demonstrating the antitumor activity of JAML agonism *in vivo*, it is interesting to speculate that agonist anti-LFA-1 antibodies may have a similar therapeutic effect for the treatment of cancer.

### CD316 and heat shock proteins

CD316 is an immunoglobulin superfamily (IgSF) protein, which associates with CD9 and CD81 within cell membranes [76], and has also been shown to localize to T cell immune synapses and interact with intracellular cytoskeleton elements to regulate T cell function [77]. CD316 has also been characterized as an early marker of dendritic cell (DC) activation and was shown to bind HSPA8, which enhanced CCL21-dependent DC migration [78]. In recent work on the function of LFA-1 for DETC function, HSPA8 was also identified as a tissue stress ligand-induced by wounding of both mouse and human skin tissue [73]. Surface expression of HSPA8 on keratinocytes was significantly increased in wounded mouse skin, and *in vitro* costimulation of DETC with anti-CD3 and recombinant HSPA8 significantly increased DETC proliferation, CD25 expression, and IL-2 production [73]. Conversely, siRNA-mediated knockdown of HSPA8 in keratinocytes resulted in decreased DETC activation after co-culture [73]. Additionally, DETC constitutively express CD316 and can be co-stimulated by an agonist anti-CD316 antibody. Therefore, CD316-HSPA8 interactions are a novel receptor-ligand pair that activates DETC in mouse skin.

Although a role for CD316-HSPA8 interactions in antitumor immunity has not been described, heat shock proteins (HSPs) have known roles in cancer and have previously been implicated in γδ T cell activation [79, 80]. HSPs are molecular chaperones that mediate protein folding and stability with diverse roles in response to cell stress associated with tissue damage, infection, and cancer [80]. In cancer, HSPs are important regulators of cell proliferation, apoptosis, migration, and metastasis [80]. Inhibitors of both HSP70 and HSP90 can inhibit the growth of a wide range of tumor cell lines *in vitro* and have shown efficacy in preclinical mouse tumor models [80, 81]. As a result, these inhibitors have been tested in human clinical trials but with limited success to date [81]. More recently, the large HSPs HSP110 and glucose-regulated protein 170 (GRP170) have been tested in cancer vaccine formulations with promising results in mouse tumor models. These HSPs effectively bind tumor antigen-derived peptides and increase APC activity and promotion of antigen-specific T cell responses [82, 83]. Tumor vaccines incorporating HSPs are the subject of ongoing human clinical trials for the treatment of a wide range of both solid and hematological malignancies [84].

In addition to their diverse roles in the maintenance of protein stability and function, HSPs have been shown to activate γδ T cells in a variety of contexts and have been suggested to be γδTCR antigens [79, 85]. In human, Vγ9Vδ2 γδ T cells have been shown to kill both Daudi B cells and primary oral tumor cells *in vitro*, which can be blocked with anti-Vγ9 or anti-HSP60 antibodies [86]. HSP70 has also been implicated in γδ T cell killing of Eppstein Barr virus-transformed B cells [87]. Mouse lymphoid γδ T cells have been shown to respond to peptides derived from HSP60 *in vivo* [88] and full-length HSP65 protein *in vitro* [89]. Additionally, mouse gut IEL γδ T cell clones proliferate and secrete cytokines after co-culture with HSP71 derived from *Mycobacterium tuberculosis in vitro* [90]. HSP60 has also been implicated in mouse γδ T cell killing of inflammatory macrophage during bacterial infection, which limits excessive inflammation [91, 92]. Together, these studies point to a model where HSPs act as a cellular stress signal in response to infection and cancer that activates γδ T cells to maintain host tissue homeostasis [79]. Despite these findings, the exact role of HSPs in γδ T cell biology has remained controversial given the ability of HSPs to bind endotoxin and studies that have shown removal of endotoxin from HSPs can limit γδ T cell activation in certain cases [93, 94]. However, given increased expression of HSPs on the surface of cancer cells compared to normal cells, it is possible that interactions such as CD316-HSPA8 are also important for γδ T cell function within tumors.

### Targeting γδ T cells for cancer immunotherapy

Due to their inherent antitumor cytotoxicity and activity independent of MHC antigen presentation, γδ T cells are a promising target for improved cancer immunotherapy. To date, the clinical use of γδ T cell-based immunotherapies has revolved around targeting Vγ9Vδ2 T cell activation via phosphoantigen sensing. Phosphoantigens such as endogenous isopentenyl pyrophosphate (IPP) and the microbial metabolite (*E*)-4-hydroxy-3-methyl-but-2-enyl-pyrophosphate (HMB-PP) drive conformational changes in the accessory molecules butyrophilin subfamily 3 member A1 (BTN3A1) and BTN2A1, which are then recognized by Vγ9Vδ2 T cells [95, 96]. The roles of butyrophilin molecules in humans and butyrophilin-like molecules in both humans and mice for γδTCR antigen recognition have been reviewed in detail elsewhere [97–99]. Although the precise mechanism of Vγ9Vδ2 T cell by butyrophilins is still an open area of investigation, Vγ9Vδ2 T cells have clear roles in antitumor immunity [100]. For immunotherapy approaches, Vγ9Vδ2 T cell activation can be targeted *in vivo* using amino-bisphosphonates, such as zoledronate, which are a class of osteoclastic bone resorption inhibitors used for the clinical treatment of osteoporosis. These drugs additionally inhibit the mevalonate pathway resulting in increased intracellular concentrations of IPP allowing for Vγ9Vδ2 T cell activation and expansion *in vivo*. Alternatively, Vγ9Vδ2 T cells can be expanded *ex vivo* using either amino-bisphosphonates or often phosphoantigens such as IPP or HMB-PP directly for ACT approaches [95, 101]. Vγ9Vδ2 T cells expanded in this way have shown enhanced antitumor activity both *in vitro* and in preclinical mouse tumor xenograft models [102]. Clinical trials using expanded Vγ9Vδ2 T cells have had limited success to date at most inducing partial responses [103], but much work is still devoted to improving *ex vivo* expansion methods for the ACT. For example, the addition of vitamin C (*L*-ascorbic acid) derivatives to bisphosphonate-driven Vγ9Vδ2 T cell expansion methods enhances their cellular expansion, cytokine production, and metabolic function [104]. Direct targeting of butyrophilin molecules *in vivo*, without the need for *ex vivo* γδ T cell expansion, is also being explored as a therapeutic strategy [96, 105, 106].

Although there is much promise in targeting γδ T cells for antitumor immunity, these therapeutic approaches will additionally have to consider the potential immunosuppressive roles of γδ T cells that can occur after activation through TCR signals. For example, phosphoantigen-activated γδ T cells have been shown to limit αβ T cells responses via expression of programmed cell death ligand 1 (PD-L1) and in the context of cancer vaccination with IL-12-secreting DCs [107, 108]. Furthermore, immunosuppressive FOXP3^+^ Vδ2^+^ T cells can be generated *in vitro* following TCR activation with IPP in the presence of IL-15 and transforming growth factor-β1 (TGF-β1) [109]. Together with other studies that have demonstrated immunosuppressive roles of γδ T cells [110] and potential protumor functions such as IL-17 production [61], these results highlight a critical limitation to overcome in the design of γδ T cell-targeted cancer immunotherapies.

Despite the potential to generate immunosuppressive γδ T cell responses, several other approaches that do not rely on phosphoantigen sensing to exploit γδ T cells for cancer immunotherapy have shown promise in preclinical studies. A protocol for the rapid expansion of highly functional Vδ1^+^ T cells from human peripheral blood has been developed [111]. These cells, termed Delta One T (DOT) cells, have high expression of NK cytotoxicity receptors, such as NKp30 and NKp44, and exhibit enhanced killing of both chronic lymphocytic leukemia (CLL) and acute myeloid leukemia (AML) cells [111, 112]. The potential roles for these NK cell receptors on γδ T cell activity have been reviewed elsewhere [31]. Because of their potential use as an allogeneic, “off-the-shelf” ACT therapy, other groups have explored γδ T cells as chimeric antigen receptor (CAR)-T cell vectors [113–115]. Additionally, bi-specific antibodies engineered to co-engage Vγ9 and tumor antigens, such as human epidermal growth factor receptor 2 (HER2) for solid tumors and CD123 for AML, are also in preclinical development [116–118].

**Figure 2.**
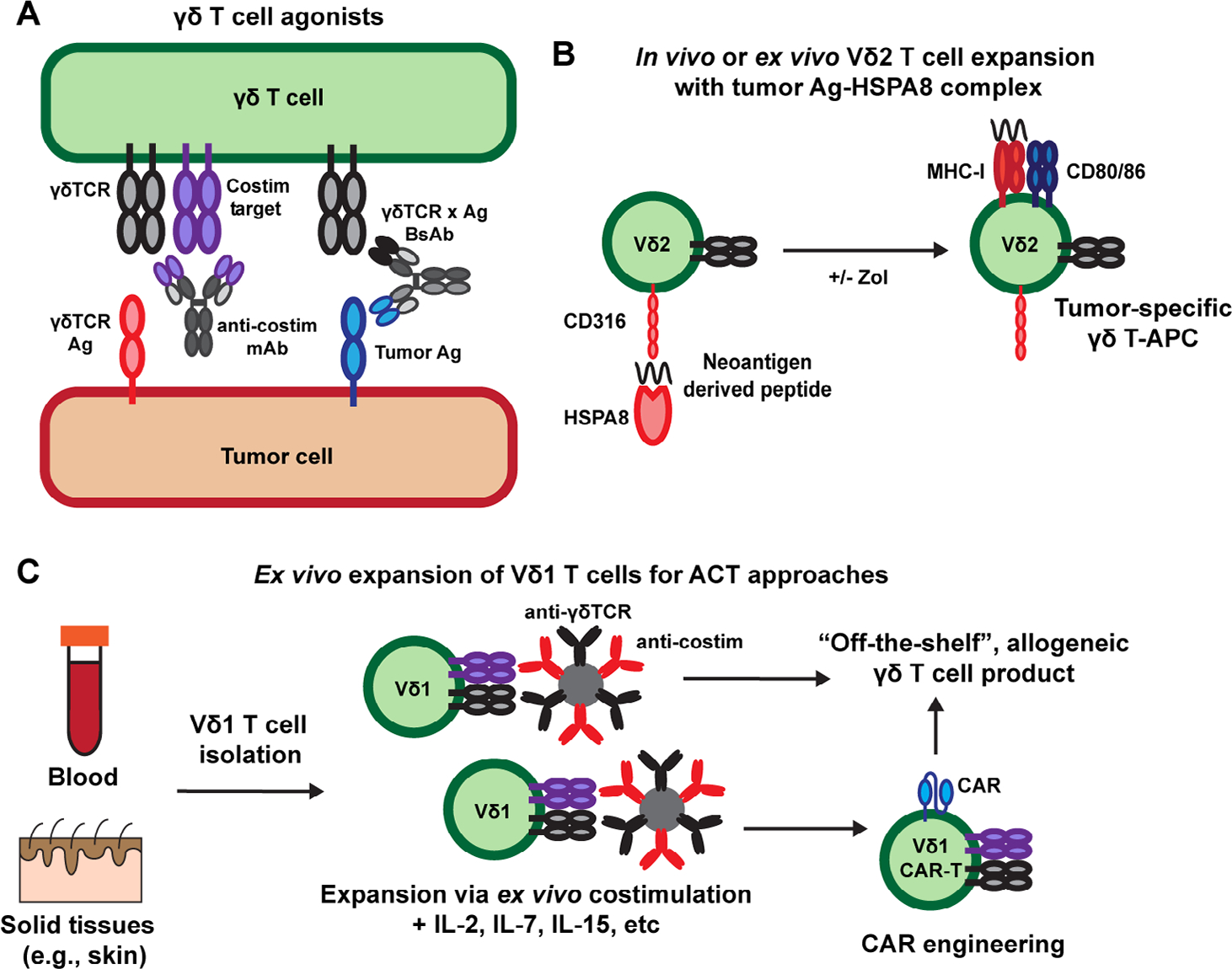
Targeting γδ T cells for cancer immunotherapy. A) γδ T cell agonists include monoclonal antibodies (mAb) and γδTCR x tumor antigen (Ag) bi-specific antibodies (BsAb) that can be used alone, in combination, or after γδ ACT therapy; B) HSPs such as HSPA8 can be used to target tumor neoantigens for uptake by Vγ9Vδ2 T cells to promote their APC function and promotion of broader antitumor immunity. HSP-based vaccines can be combined with Vγ9Vδ2 T cell expansion using bisphosphonate drugs such as zoledronate (Zol) either *ex vivo* for ACT therapies or directly *in vivo*; C) addition of costimulatory signals (e.g., with antibody-coated beads) during *ex vivo* expansion of Vδ1 T cells can be used to generate more functional allogeneic T cell products for the ACT and CAR-T cell approaches

Targeting γδ T cell costimulatory molecules is potentially a novel approach to build upon these current strategies being explored to leverage the antitumor activity of γδ T cells. Of note, many groups have developed agonist monoclonal antibodies targeting other T cell costimulatory molecules such as 4–1BB [CD137, tumor necrosis factor superfamily (TNFRSF) member 9], OX40 (CD134, TNFRSF4), CD28, and ICOS (CD278, inducible T cell costimulator), and many of these agents are currently being tested in human clinal trials [119]. Unlike antagonistic anti-PD-1 and anti-cytotoxic T lymphocyte associated protein 4 (anti-CTLA-4) that limit T cell inhibition by block receptor-ligand interactions, these agonist antibodies mediate their effects by clustering costimulatory molecules on the surface of T cells to induce intracellular signaling events that potentiate T cell activation [119, 120]. Despite much work in this area, T cell agonist monoclonal antibodies have had limited success in clinical trials to date, which may be associated with the limited ability of typical bivalent monoclonal antibodies to mediate this receptor clustering [119]. Similar challenges would likely apply to the clinical development of agonist antibodies against the γδ T cell costimulatory molecules discussed above. However, novel engineering approaches such as the use of highly multivalent ligand-antibody fusions and Fc receptor engineering approaches to enhance cross-linking of therapeutic antibodies *in trans* may overcome these challenges [119, 121, 122]. Additionally, agonist monoclonal antibodies against γδ T cell targets are potentially a useful way to improve the efficacy of either γδTCR x tumor antigen bi-specific antibodies or γδ T cell ACT therapies when used in combination (Figure 2A).

In addition to representing potential targets of agonist monoclonal antibody therapies, CD100 and CD316 have unique roles outside of γδ T cell costimulation that may represent intriguing targets for immunotherapy. Although CD100 has been described as a costimulatory ligand for DETC, its role in cancer appears to be mostly pro-tumorigenic as demonstrated by the antitumor activity of blocking anti-CD100 antibodies in mouse tumor models [52, 59]. Based on these studies, an antagonistic anti-human CD100 antibody (pepinemab) is currently being tested in phase II clinical trials for the treatment of human cancers [123, 124]. If successful in clinical trials, the effect of blocking anti-CD100 antibodies on inhibition of pro-tumor IL-17-producing γδ T cells *versus* antitumor IFNγ-producing γδ T cells will be of interest and may help inform which solid tumor indications are most suitable for treatment [61]. Similarly, CD316 is not specific to γδ T cell function and has been shown to induce DC activation upon binding to HSPA8 [78]. Given that HSPs have been used in tumor vaccine formulations and that human Vγ9Vδ2 T cells possess antigen-presenting capabilities, it is interesting to speculate that HSPA8-derived tumor vaccines, delivered directly *in vivo* or in combination with *ex vivo* expanded Vγ9Vδ2 T cells for ACT therapy, may be a novel approach to activate Vγ9Vδ2 T cell APC function and stimulate endogenous antigen-specific αβ T cells responses [80, 125–127] (Figure 2B). Importantly, recent evidence suggests that Vγ9Vδ2 T cells can both kill tumor targets and cross-present tumor antigens [128]. Whether the use of HSPs and targeting CD316 is a relevant strategy to exploit this activity remains to be explored.

As discussed above, the use of γδ T cells in ACT therapies has garnered much interest because of their potential use as an “off-the-self”, allogeneic T cell therapy without the need for donor-recipient MHC matching [95]. The use of bisphosphonates to expand Vγ9Vδ2 T cells and novel protocols to expand Vδ1^+^ T cells may prove to be effective immunotherapies, but there is likely much room for improvement in the engineering of these T cell products. One challenge with the development of γδ T cell-based ACT therapies is the limited ability to activate and expand cells *ex vivo* with more standard T cell costimulatory molecules such as CD28, which are critical for the manufacturing of αβ T cell-based cellular therapies [129, 130]. Vγ9Vδ2 T cells typically express much lower levels of CD28 compared to αβ T cells and decrease expression upon activation, which may limit the use of CD28 costimulation in Vγ9Vδ2 T cell expansion [131–134]. Furthermore, tissue-resident Vδ1^+^ T cells in humans may be the best population to utilize in ACT therapy based on their intrinsic ability to sense signals of self-stress due to tissue damage and malignancy and function within non-lymphoid tissues. However, research on mouse tissue-resident γδ T cells, such as DETC, has found that these subsets typically lack expression of CD28 and therefore require other signals for activation, which may also apply to human Vδ1^+^ T cells [1]. Thus, costimulation of either Vγ9Vδ2 T cells or tissue-derived Vδ1^+^ T cells via NKG2D, JAML, CD100, LFA-1, or CD316 may be novel approach to expand these cells *ex vivo* (Figure 2C). Similar to the development of DOT cell expansion protocols [111], this work would have to be done empirically and may require sequential activation steps utilizing one or more of these costimulatory ligands at a time. If such a strategy is viable, γδ T cells expanded in this way may be ideal candidates for ACT approaches including CAR-T engineering.

## Conclusion

Basic research interrogating γδ T cell biology in mice has identified an important role for γδ T cells in tumor surveillance and led to the identification of novel costimulatory ligands that control their activation within non-lymphoid tissues. Despite the evolutionary divergence of mouse and human γδ T cells, these findings in mice have potential implications for the treatment of human cancers. Most notably, γδ T cell-mediated tumor surveillance and cytotoxicity mediated by NKG2D has clear parallels in mouse and human, and studies of NKG2D’s function on γδ T cells have helped lead to the development of agents targeting NKG2D that are currently being tested in human clinical trials [135]. Although less well characterized, other γδ T cell costimulatory ligands such as JAML, CD100, LFA-1, and CD316 may also represent important targets for cancer immunotherapy. Future studies of these costimulatory molecules and the identification of additional novel mechanisms of γδ T cell activation will be important to leverage the unique properties of these cells for successful cancer immunotherapy interventions.

## Figures and Tables

**Table 1.** Most common γδ T cell subsets in mouse and human

	Circulating subsets	Tissue-resident subsets

Mouse	Vγ1.1^+^	Vγ3^+^ (epidermis)
	Vγ2^+^	Vγ4^+^ (dermis, lung, uterus, adipose, liver)
		Vγ5^+^ (gut)
Human	Vδ2^+^	Vδ1^+^ (epidermis, dermis, adipose, gut, liver)

*Note.* Adapted from “γδ T cells in cancer [12]” by Silva-Santos B, Serre K, Norell H. Nat Rev Immunol. 2015;15:683–91 (https://pubmed.ncbi.nlm.nih.gov/26449179/). © 2015 Macmillan Publishers Limited.

## Abbreviations

ACT: adoptive cell transfer
APCs: antigen-presenting cells
CAR: chimeric antigen receptor
CXADR: coxsackie and adenovirus receptor
DAP10/12: DNAX-activating protein 10/12
DC: dendritic cell
DETC: dendritic epidermal T cells
H60: histocompatibility 60
HSPs: heat shock proteins
HSPA8: heat shock protein family A member 8
ICAM-1: intercellular adhesion molecule-1
IFNγ: interferon-γ
IL-17: interleukin-17
IPP: isopentenyl pyrophosphate
JAML: junctional adhesion molecule-like protein
KGF-1: keratinocyte growth factor-1
LFA-1: lymphocyte function-associated antigen 1
MICA/B: major histocompatibility complex class I polypeptide-related sequence A/B
MHC: major histocompatibility complex
PI3K: phosphoinositide 3-kinase
NK: natural killer
NKG2D: natural killer group 2D
Rae-1: retinoic acid early inducible 1
TCR: T cell receptor
*Tcrd^−/−^*: T cell receptor delta-deficient
TIL: tumor-infiltrating lymphocytes
TME: tumor microenvironment

## References

[1] Nielsen MM, Witherden DA, Havran WL. γδ T cells in homeostasis and host defence of epithelial barrier tissues. Nat Rev Immunol. 2017;17:733–45. 28920588 10.1038/nri.2017.101 PMC5771804

[2] Girardi M, Oppenheim DE, Steele CR, Lewis JM, Glusac E, Filler R, Regulation of cutaneous malignancy by gammadelta T cells. Science. 2001;294:605–9. 11567106 10.1126/science.1063916

[3] Gao Y, Yang W, Pan M, Scully E, Girardi M, Augenlicht LH, Gamma delta T cells provide an early source of interferon gamma in tumor immunity. J Exp Med. 2003;198:433–42. 12900519 10.1084/jem.20030584 PMC2194096

[4] Lança T, Costa MF, Gonçalves-Sousa N, Rei M, Grosso AR, Penido C, Protective role of the inflammatory CCR2/CCL2 chemokine pathway through recruitment of type 1 cytotoxic γδ T lymphocytes to tumor beds. J Immunol. 2013;190:6673–80. 23686489 10.4049/jimmunol.1300434

[5] Silva-Santos B, Mensurado S, Coffelt SB. gamma delta T cells: pleiotropic immune effectors with therapeutic potential in cancer. Nat Rev Cancer. 2019;19:392–404. 31209264 10.1038/s41568-019-0153-5 PMC7614706

[6] Gentles AJ, Newman AM, Liu CL, Bratman SV, Feng W, Kim D, The prognostic landscape of genes and infiltrating immune cells across human cancers. Nat Med. 2015;21:938–45. 26193342 10.1038/nm.3909 PMC4852857

[7] Gherardin NA, Waldeck K, Caneborg A, Martelotto LG, Balachander S, Zethoven M, γδ T cells in merkel cell carcinomas have a proinflammatory profile prognostic of patient survival. Cancer Immunol Res. 2021;9:612–23. 33674358 10.1158/2326-6066.CIR-20-0817

[8] Wu Y, Kyle-Cezar F, Woolf RT, Naceur-Lombardelli C, Owen J, Biswas D, An innate-like Vδ1^+^ γδ T cell compartment in the human breast is associated with remission in triple-negative breast cancer. Sci Transl Med. 2019;11:eaax9364. 31597756 10.1126/scitranslmed.aax9364 PMC6877350

[9] Foord E, Arruda LCM, Gaballa A, Klynning C, Uhlin M. Characterization of ascites- and tumor-infiltrating γδ T cells reveals distinct repertoires and a beneficial role in ovarian cancer. Sci Transl Med. 2021;13:eabb0192. 33472952 10.1126/scitranslmed.abb0192

[10] Imbert C, Olive D. γδ T cells in tumor microenvironment. Adv Exp Med Biol. 2020;1273:91–104. 33119877 10.1007/978-3-030-49270-0_5

[11] Willcox BE, Willcox CR. γδ TCR ligands: the quest to solve a 500-million-year-old mystery. Nat Immunol. 2019;20:121–8. 30664765 10.1038/s41590-018-0304-y

[12] Silva-Santos B, Serre K, Norell H. γδ T cells in cancer. Nat Rev Immunol. 2015;15:683–91. 26449179 10.1038/nri3904

[13] Thelen F, Witherden DA. Get in touch with dendritic epithelial T cells! Front Immunol. 2020;11:1656. 32849572 10.3389/fimmu.2020.01656 PMC7403176

[14] Jameson J, Ugarte K, Chen N, Yachi P, Fuchs E, Boismenu R, A role for skin gammadelta T cells in wound repair. Science. 2002;296:747–9. 11976459 10.1126/science.1069639

[15] Komori HK, Witherden DA, Kelly R, Sendaydiego K, Jameson JM, Teyton L, Cutting edge: dendritic epidermal γδ T cell ligands are rapidly and locally expressed by keratinocytes following cutaneous wounding. J Immunol. 2012;188:2972–6. 22393149 10.4049/jimmunol.1100887 PMC3311739

[16] Sharp LL, Jameson JM, Cauvi G, Havran WL. Dendritic epidermal T cells regulate skin homeostasis through local production of insulin-like growth factor 1. Nat Immunol. 2005;6:73–9. 15592472 10.1038/ni1152

[17] Matsue H, Cruz PD Jr, Bergstresser PR, Takashima A. Profiles of cytokine mRNA expressed by dendritic epidermal T cells in mice. J Invest Dermatol. 1993;101:537–42. 8409520 10.1111/1523-1747.ep12365917

[18] Witherden DA, Johnson MD, Havran WL. Coreceptors and their ligands in epithelial γδ T cell biology. Front Immunol. 2018;9:731. 29686687 10.3389/fimmu.2018.00731 PMC5900413

[19] Baugh R, Khalique H, Seymour LW. Convergent evolution by cancer and viruses in evading the NKG2D immune response. Cancers (Basel). 2020;12:3827. 10.3390/cancers12123827 PMC7766243 33352921

[20] Lanier LL. NKG2D receptor and its ligands in host defense. Cancer Immunol Res. 2015;3:575–82. 26041808 10.1158/2326-6066.CIR-15-0098 PMC4457299

[21] Bauer S, Groh V, Wu J, Steinle A, Phillips JH, Lanier LL, Activation of NK cells and T cells by NKG2D, a receptor for stress-inducible MICA. Science. 1999;285:727–9. 10426993 10.1126/science.285.5428.727

[22] Carena I, Shamshiev A, Donda A, Colonna M, Libero GD. Major histocompatibility complex class I molecules modulate activation threshold and early signaling of T cell antigen receptor-gamma/delta stimulated by nonpeptidic ligands. J Exp Med. 1997;186:1769–74. 9362537 10.1084/jem.186.10.1769 PMC2199143

[23] Strid J, Roberts SJ, Filler RB, Lewis JM, Kwong BY, Schpero W, Acute upregulation of an NKG2D ligand promotes rapid reorganization of a local immune compartment with pleiotropic effects on carcinogenesis. Nat Immunol. 2008;9:146–54. 18176566 10.1038/ni1556

[24] Dalessandri T, Crawford G, Hayes M, Castro Seoane R, Strid J. IL-13 from intraepithelial lymphocytes regulates tissue homeostasis and protects against carcinogenesis in the skin. Nat Commun. 2016;7:12080. 27357235 10.1038/ncomms12080 PMC4931319

[25] Strid J, Sobolev O, Zafirova B, Polic B, Hayday A. The intraepithelial T cell response to NKG2D-ligands links lymphoid stress surveillance to atopy. Science. 2011;334:1293–7. 22144628 10.1126/science.1211250 PMC3842529

[26] Yoshida S, Mohamed RH, Kajikawa M, Koizumi J, Tanaka M, Fugo K, Involvement of an NKG2D ligand H60c in epidermal dendritic T cell-mediated wound repair. J Immunol. 2012;188:3972–9. 22403443 10.4049/jimmunol.1102886

[27] Nielsen MM, Dyring-Andersen B, Schmidt JD, Witherden D, Lovato P, Woetmann A, NKG2D-dependent activation of dendritic epidermal T cells in contact hypersensitivity. J Invest Dermatol. 2015;135:1311–9. 25634359 10.1038/jid.2015.23 PMC4402141

[28] Nitahara A, Shimura H, Ito A, Tomiyama K, Ito M, Kawai K. NKG2D ligation without T cell receptor engagement triggers both cytotoxicity and cytokine production in dendritic epidermal T cells. J Invest Dermatol. 2006;126:1052–8. 16484989 10.1038/sj.jid.5700112

[29] Ibusuki A, Kawai K, Yoshida S, Uchida Y, Nitahara-Takeuchi A, Kuroki K, NKG2D triggers cytotoxicity in murine epidermal γδ T cells via PI3K-dependent, Syk/ZAP70-independent signaling pathway. J Invest Dermatol. 2014;134:396–404. 23962808 10.1038/jid.2013.353

[30] Wrobel P, Shojaei H, Schittek B, Gieseler F, Wollenberg B, Kalthoff H, Lysis of a broad range of epithelial tumour cells by human gamma delta T cells: involvement of NKG2D ligands and T-cell receptor- *versus* NKG2D-dependent recognition. Scand J Immunol. 2007;66:320–8. 17635809 10.1111/j.1365-3083.2007.01963.x

[31] Silva-Santos B, Strid J. Working in “NK mode”: natural killer group 2 member D and natural cytotoxicity receptors in stress-surveillance by γδ T cells. Front Immunol. 2018;9:851. 29740448 10.3389/fimmu.2018.00851 PMC5928212

[32] Cao G, Wang Q, Li G, Meng Z, Liu H, Tong J, mTOR inhibition potentiates cytotoxicity of Vγ4 γδ T cells via up-regulating NKG2D and TNF-α. J Leukoc Biol. 2016;100:1181–9. 27256566 10.1189/jlb.5A0116-053RR

[33] Kummer D, Ebnet K. Junctional adhesion molecules (JAMs): the JAM-integrin connection. Cells. 2018;7:25. 10.3390/cells7040025 PMC5946102 29587442

[34] Lauko A, Mu Z, Gutmann DH, Naik UP, Lathia JD. Junctional adhesion molecules in cancer: a paradigm for the diverse functions of cell-cell interactions in tumor progression. Cancer Res. 2020;80:4878–85. 32816855 10.1158/0008-5472.CAN-20-1829 PMC7669553

[35] Luissint AC, Lutz PG, Calderwood DA, Couraud PO, Bourdoulous S. JAM-L-mediated leukocyte adhesion to endothelial cells is regulated in cis by alpha4beta1 integrin activation. J Cell Biol. 2008;183:1159–73. 19064666 10.1083/jcb.200805061 PMC2600739

[36] Moog-Lutz C, Cavé-Riant F, Guibal FC, Breau MA, Di Gioia Y, Couraud PO, JAML, a novel protein with characteristics of a junctional adhesion molecule, is induced during differentiation of myeloid leukemia cells. Blood. 2003;102:3371–8. 12869515 10.1182/blood-2002-11-3462

[37] Witherden DA, Verdino P, Rieder SE, Garijo O, Mills RE, Teyton L, The junctional adhesion molecule JAML is a costimulatory receptor for epithelial gammadelta T cell activation. Science. 2010;329:1205–10. 20813954 10.1126/science.1192698 PMC2943937

[38] McGraw JM, Thelen F, Hampton EN, Bruno NE, Young TS, Havran WL, JAML promotes CD8 and γδ T cell antitumor immunity and is a novel target for cancer immunotherapy. J Exp Med. 2021;218:e20202644. 34427588 10.1084/jem.20202644 PMC8404475

[39] Reeh M, Bockhorn M, Görgens D, Vieth M, Hoffmann T, Simon R, Presence of the coxsackievirus and adenovirus receptor (CAR) in human neoplasms: a multitumour array analysis. Br J Cancer. 2013;109:1848–58. 24022195 10.1038/bjc.2013.509 PMC3790165

[40] Nilchian A, Johansson J, Ghalali A, Asanin ST, Santiago A, Rosencrantz O, CXADR-mediated formation of an AKT inhibitory signalosome at tight junctions controls epithelial-mesenchymal plasticity in breast cancer. Cancer Res. 2019;79:47–60. 30385615 10.1158/0008-5472.CAN-18-1742

[41] Anders M, Vieth M, Röcken C, Ebert M, Pross M, Gretschel S, Loss of the coxsackie and adenovirus receptor contributes to gastric cancer progression. Br J Cancer. 2009;100:352–9. 19142187 10.1038/sj.bjc.6604876 PMC2634721

[42] Maleki KT, Cornillet M, Björkström NK. Soluble SEMA4D/CD100: a novel immunoregulator in infectious and inflammatory diseases. Clin Immunol. 2016;163:52–9. 26732857 10.1016/j.clim.2015.12.012

[43] Shi W, Kumanogoh A, Watanabe C, Uchida J, Wang X, Yasui T, The class IV semaphorin CD100 plays nonredundant roles in the immune system: defective B and T cell activation in CD100-deficient mice. Immunity. 2000;13:633–42. 11114376 10.1016/s1074-7613(00)00063-7

[44] Kumanogoh A, Suzuki K, Ch’ng E, Watanabe C, Marukawa S, Takegahara N, Requirement for the lymphocyte semaphorin, CD100, in the induction of antigen-specific T cells and the maturation of dendritic cells. J Immunol. 2002;169:1175–81. 12133937 10.4049/jimmunol.169.3.1175

[45] Pan C, Baumgarth N, Parnes JR. CD72-deficient mice reveal nonredundant roles of CD72 in B cell development and activation. Immunity. 1999;11:495–506. 10549631 10.1016/s1074-7613(00)80124-7

[46] Elhabazi A, Delaire S, Bensussan A, Boumsell L, Bismuth G. Biological activity of soluble CD100. I. The extracellular region of CD100 is released from the surface of T lymphocytes by regulated proteolysis. J Immunol. 2001;166:4341–7. 11254687 10.4049/jimmunol.166.7.4341

[47] Delaire S, Billard C, Tordjman R, Chédotal A, Elhabazi A, Bensussan A, Biological activity of soluble CD100. II. Soluble CD100, similarly to H-SemaIII, inhibits immune cell migration. J Immunol. 2001;166:4348–54. 11254688 10.4049/jimmunol.166.7.4348

[48] Kruger RP, Aurandt J, Guan KL. Semaphorins command cells to move. Nat Rev Mol Cell Biol. 2005;6:789–800. 16314868 10.1038/nrm1740

[49] Witherden DA, Watanabe M, Garijo O, Rieder SE, Sarkisyan G, Cronin SJ, The CD100 receptor interacts with its plexin B2 ligand to regulate epidermal γδ T cell function. Immunity. 2012;37:314–25. 22902232 10.1016/j.immuni.2012.05.026 PMC3430606

[50] Meehan TF, Witherden DA, Kim CH, Sendaydiego K, Ye I, Garijo O, Protection against colitis by CD100-dependent modulation of intraepithelial γδ T lymphocyte function. Mucosal Immunol. 2014;7:134–42. 23695512 10.1038/mi.2013.32 PMC3795871

[51] Zhang C, Xiao C, Dang E, Cao J, Zhu Z, Fu M, CD100-plexin-B2 promotes the inflammation in psoriasis by activating NF-κB and the inflammasome in keratinocytes. J Invest Dermatol. 2018;138:375–83. 28927892 10.1016/j.jid.2017.09.005

[52] Tamagnone L, Franzolin G. Targeting semaphorin 4D in cancer: a look from different perspectives. Cancer Res. 2019;79:5146–8. 31615809 10.1158/0008-5472.CAN-19-2387

[53] Wang JS, Jing CQ, Shan KS, Chen YZ, Guo XB, Cao ZX, Semaphorin 4D and hypoxia-inducible factor-1α overexpression is related to prognosis in colorectal carcinoma. World J Gastroenterol. 2015;21:2191–8. 25717256 10.3748/wjg.v21.i7.2191 PMC4326158

[54] Chen Y, Zhang L, Lv R, Zhang WQ. Overexpression of semaphorin4D indicates poor prognosis and prompts monocyte differentiation toward M2 macrophages in epithelial ovarian cancer. Asian Pac J Cancer Prev 2013;14:5883–90. 24289594 10.7314/apjcp.2013.14.10.5883

[55] Ch’ng E, Tomita Y, Zhang B, He J, Hoshida Y, Qiu Y, Prognostic significance of CD100 expression in soft tissue sarcoma. Cancer. 2007;110:164–72. 17520683 10.1002/cncr.22764

[56] Liu H, Yang Y, Xiao J, Yang S, Liu Y, Kang W, Semaphorin 4D expression is associated with a poor clinical outcome in cervical cancer patients. Microvasc Res. 2014;93:1–8. 24603190 10.1016/j.mvr.2014.02.007

[57] Sierra JR, Corso S, Caione L, Cepero V, Conrotto P, Cignetti A, Tumor angiogenesis and progression are enhanced by Sema4D produced by tumor-associated macrophages. J Exp Med. 2008;205:1673–85. 18559453 10.1084/jem.20072602 PMC2442644

[58] Younis RH, Han KL, Webb TJ. Human head and neck squamous cell carcinoma-associated semaphorin 4D induces expansion of myeloid-derived suppressor cells. J Immunol. 2016;196:1419–29. 26740106 10.4049/jimmunol.1501293 PMC4722498

[59] Clavijo PE, Friedman J, Robbins Y, Moore EC, Smith E, Zauderer M, Semaphorin4D inhibition improves response to immune-checkpoint blockade via attenuation of MDSC recruitment and function. Cancer Immunol Res. 2019;7:282–91. 30514791 10.1158/2326-6066.CIR-18-0156 PMC8326929

[60] Wang HM, Zhang XH, Ye LQ, Zhang K, Yang NN, Geng S, Insufficient CD100 shedding contributes to suppression of CD8^+^ T-cell activity in non-small cell lung cancer. Immunology. 2020;160:209–19. 32149403 10.1111/imm.13189 PMC7218665

[61] Chitadze G, Oberg HH, Wesch D, Kabelitz D. The ambiguous role of γδ T lymphocytes in antitumor immunity. Trends Immunol. 2017;38:668–78. 28709825 10.1016/j.it.2017.06.004

[62] Bui TM, Wiesolek HL, Sumagin R. ICAM-1: a master regulator of cellular responses in inflammation, injury resolution, and tumorigenesis. J Leukoc Biol. 2020;108:787–99. 32182390 10.1002/JLB.2MR0220-549R PMC7977775

[63] Walling BL, Kim M. LFA-1 in T cell migration and differentiation. Front Immunol. 2018;9:952. 29774029 10.3389/fimmu.2018.00952 PMC5943560

[64] Perez OD, Mitchell D, Jager GC, South S, Murriel C, McBride J, Leukocyte functional antigen 1 lowers T cell activation thresholds and signaling through cytohesin-1 and Jun-activating binding protein 1. Nat Immunol. 2003;4:1083–92. 14528303 10.1038/ni984

[65] Goedegebuure PS, Braakman E, Segal DM, Vreugdenhil RJ, Bolhuis RL. Lymphocyte leukocyte function-associated antigen 1 interacting with target cell intercellular adhesion molecule 1 co-activates cytolysis triggered via CD16 or the receptor involved in major histocompatibility antigen-unrestricted lysis. Int Immunol. 1990;2:1213–20. 1982501 10.1093/intimm/2.12.1213

[66] Ensslin AS, Formby B. Comparison of cytolytic and proliferative activities of human gamma delta and alpha beta T cells from peripheral blood against various human tumor cell lines. J Natl Cancer Inst. 1991;83:1564–9. 1683671 10.1093/jnci/83.21.1564

[67] Nelson EL, Kim HT, Mar ND, Goralski TJ, Mclntyre BW, Clayberger C, Novel tumor-associated accessory molecules involved in the gamma/delta cytotoxic T-lymphocyte-Burkitt’s lymphoma interaction. Cancer. 1995;75:886–93. 7530169 10.1002/1097-0142(19950201)75:3<886::aid-cncr2820750321>3.0.co;2-g

[68] Uchida R, Ashihara E, Sato K, Kimura S, Kuroda J, Takeuchi M, Gamma delta T cells kill myeloma cells by sensing mevalonate metabolites and ICAM-1 molecules on cell surface. Biochem Biophys Res Commun. 2007;354:613–8. 17250803 10.1016/j.bbrc.2007.01.031

[69] Liu Z, Guo B, Lopez RD. Expression of intercellular adhesion molecule (ICAM)-1 or ICAM-2 is critical in determining sensitivity of pancreatic cancer cells to cytolysis by human gammadelta-T cells: implications in the design of gammadelta-T-cell-based immunotherapies for pancreatic cancer. J Gastroenterol Hepatol. 2009;24:900–11. 19175829 10.1111/j.1440-1746.2008.05668.x

[70] Weng RR, Lu HH, Lin CT, Fan CC, Lin RS, Huang TC, Epigenetic modulation of immune synaptic-cytoskeletal networks potentiates γδ T cell-mediated cytotoxicity in lung cancer. Nat Commun. 2021;12:2163. 33846331 10.1038/s41467-021-22433-4 PMC8042060

[71] Thomas ML, Badwe RA, Deshpande RK, Samant UC, Chiplunkar SV. Role of adhesion molecules in recruitment of Vdelta1 T cells from the peripheral blood to the tumor tissue of esophageal cancer patients. Cancer Immunol Immunother. 2001;50:218–25. 11459174 10.1007/s002620100190 PMC11036823

[72] Byeseda SE, Burns AR, Dieffenbaugher S, Rumbaut RE, Smith CW, Li Z. ICAM-1 is necessary for epithelial recruitment of gammadelta T cells and efficient corneal wound healing. Am J Pathol. 2009;175:571–9. 19608878 10.2353/ajpath.2009.090112 PMC2716957

[73] Johnson MD, Otuki MF, Cabrini DA, Rudolph R, Witherden DA, Havran WL. Hspa8 and ICAM-1 as damage-induced mediators of γδ T cell activation. J Leukoc Biol. 2022;111:135–45. 33847413 10.1002/JLB.3AB0420-282R

[74] Nagaoka T, Kaburagi Y, Hamaguchi Y, Hasegawa M, Takehara K, Steeber DA, Delayed wound healing in the absence of intercellular adhesion molecule-1 or L-selectin expression. Am J Pathol. 2000;157:237–47. 10880393 10.1016/S0002-9440(10)64534-8 PMC1850195

[75] Sumagin R, Brazil JC, Nava P, Nishio H, Alam A, Luissint AC, Neutrophil interactions with epithelial-expressed ICAM-1 enhances intestinal mucosal wound healing. Mucosal Immunol. 2016;9:1151–62. 26732677 10.1038/mi.2015.135 PMC4935657

[76] Stipp CS, Kolesnikova TV, Hemler ME. EWI-2 is a major CD9 and CD81 partner and member of a novel Ig protein subfamily. J Biol Chem. 2001;276:40545–54. 11504738 10.1074/jbc.M107338200

[77] Gordón-Alonso M, Sala-Valdés M, Rocha-Perugini V, Pérez-Hernández D, López-Martín S, Ursa A, EWI-2 association with α-actinin regulates T cell immune synapses and HIV viral infection. J Immunol. 2012;189:689–700. 22689882 10.4049/jimmunol.1103708

[78] Kettner S, Kalthoff F, Graf P, Priller E, Kricek F, Lindley I, EWI-2/CD316 is an inducible receptor of HSPA8 on human dendritic cells. Mol Cell Biol. 2007;27:7718–26. 17785435 10.1128/MCB.00180-07 PMC2169036

[79] Hirsh MI, Junger WG. Roles of heat shock proteins and gamma delta T cells in inflammation. Am J Respir Cell Mol Biol. 2008;39:509–13. 18566334 10.1165/rcmb.2008-0090TR PMC2574523

[80] Wu J, Liu T, Rios Z, Mei Q, Lin X, Cao S. Heat shock proteins and cancer. Trends Pharmacol Sci. 2017;38:226–56. 28012700 10.1016/j.tips.2016.11.009

[81] Chatterjee S, Burns TF. Targeting heat shock proteins in cancer: a promising therapeutic approach. Int J Mol Sci. 2017;18:1978. 10.3390/ijms18091978 PMC5618627 28914774

[82] Ren F, Xu Y, Mao L, Ou R, Ding Z, Zhang X, Heat shock protein 110 improves the antitumor effects of the cytotoxic T lymphocyte epitope E7(49–57) in mice. Cancer Biol Ther. 2010;9:134–41. 19901562 10.4161/cbt.9.2.10391

[83] Manjili MH, Park JE, Facciponte JG, Wang XY, Subjeck JR. Immunoadjuvant chaperone, GRP170, induces ‘danger signals’ upon interaction with dendritic cells. Immunol Cell Biol. 2006;84:203–8. 16519738 10.1111/j.1440-1711.2006.01418.x PMC3094687

[84] Shevtsov M, Multhoff G. Heat shock protein-peptide and HSP-based immunotherapies for the treatment of cancer. Front Immunol. 2016;7:171. 27199993 10.3389/fimmu.2016.00171 PMC4850156

[85] O’Brien RL, Born W. Heat shock proteins as antigens for gamma delta T cells. Semin Immunol. 1991;3:81–7. 1832320

[86] Laad AD, Thomas ML, Fakih AR, Chiplunkar SV. Human gamma delta T cells recognize heat shock protein-60 on oral tumor cells. Int J Cancer. 1999;80:709–14. 10048972 10.1002/(sici)1097-0215(19990301)80:5<709::aid-ijc14>3.0.co;2-r

[87] Zhang H, Hu H, Jiang X, He H, Cui L, He W. Membrane HSP70: the molecule triggering γδ T cells in the early stage of tumorigenesis. Immunol Invest. 2005;34:453–68. 16302688 10.1080/08820130500265349

[88] Fu YX, Cranfill R, Vollmer M, Van Der Zee R, O’Brien RL, Born W. *In vivo* response of murine gamma delta T cells to a heat shock protein-derived peptide. Proc Natl Acad Sci U S A. 1993;90:322–6. 8093560 10.1073/pnas.90.1.322 PMC45652

[89] Tsuji M, Mombaerts P, Lefrancois L, Nussenzweig RS, Zavala F, Tonegawa S. Gamma delta T cells contribute to immunity against the liver stages of malaria in alpha beta T-cell-deficient mice. Proc Natl Acad Sci U S A. 1994;91:345–9. 8278391 10.1073/pnas.91.1.345 PMC42944

[90] Beagley KW, Fujihashi K, Black CA, Lagoo AS, Yamamoto M, McGhee JR, The *Mycobacterium tuberculosis* 71-kDa heat-shock protein induces proliferation and cytokine secretion by murine gut intraepithelial lymphocytes. Eur J Immunol. 1993;23:2049–52. 8344373 10.1002/eji.1830230852

[91] Egan PJ, Carding SR. Downmodulation of the inflammatory response to bacterial infection by gammadelta T cells cytotoxic for activated macrophages. J Exp Med. 2000;191:2145–58. 10859339 10.1084/jem.191.12.2145 PMC2193196

[92] Belles C, Kuhl A, Nosheny R, Carding SR. Plasma membrane expression of heat shock protein 60 *in vivo* in response to infection. Infect Immun. 1999;67:4191–200. 10417191 10.1128/iai.67.8.4191-4200.1999 PMC96724

[93] Habich C, Kempe K, van der Zee R, Rümenapf R, Akiyama H, Kolb H, Heat shock protein 60: specific binding of lipopolysaccharide. J Immunol. 2005;174:1298–305. 15661886 10.4049/jimmunol.174.3.1298

[94] Bausinger H, Lipsker D, Ziylan U, Manié S, Briand JP, Cazenave JP, Endotoxin-free heat-shock protein 70 fails to induce APC activation. Eur J Immunol. 2002;32:3708–13. 12516564 10.1002/1521-4141(200212)32:12<3708::AID-IMMU3708>3.0.CO;2-C

[95] Miyashita M, Shimizu T, Ashihara E, Ukimura O. Strategies to improve the antitumor effect of γδ T cell immunotherapy for clinical application. Int J Mol Sci. 2021;22:8910. 34445615 10.3390/ijms22168910 PMC8396358

[96] Rigau M, Uldrich AP, Behren A. Targeting butyrophilins for cancer immunotherapy. Trends Immunol. 2021;42:670–80. 34253468 10.1016/j.it.2021.06.002

[97] Uldrich AP, Rigau M, Godfrey DI. Immune recognition of phosphoantigen-butyrophilin molecular complexes by γδ T cells. Immunol Rev. 2020;298:74–83. 33017054 10.1111/imr.12923

[98] Ribot JC, Lopes N, Silva-Santos B. γδ T cells in tissue physiology and surveillance. Nat Rev Immunol. 2021;21:221–32. 33057185 10.1038/s41577-020-00452-4

[99] Vantourout P, Laing A, Woodward MJ, Zlatareva I, Apolonia L, Jones AW, Heteromeric interactions regulate butyrophilin (BTN) and BTN-like molecules governing γδ T cell biology. Proc Natl Acad Sci U S A. 2018;115:1039–44. 29339503 10.1073/pnas.1701237115 PMC5798315

[100] Hayday AC. γδ T cell update: adaptate orchestrators of immune surveillance. J Immunol. 2019;203:311–20. 31285310 10.4049/jimmunol.1800934

[101] Kunzmann V, Bauer E, Feurle J, Weissinger F, Tony HP, Wilhelm M. Stimulation of gammadelta T cells by aminobisphosphonates and induction of antiplasma cell activity in multiple myeloma. Blood. 2000;96:384–92. 10887096

[102] Yazdanifar M, Barbarito G, Bertaina A, Airoldi I. γδ T cells: the ideal tool for cancer immunotherapy. Cells. 2020;9:1305. 10.3390/cells9051305 PMC7290982 32456316

[103] Fisher JP, Heuijerjans J, Yan M, Gustafsson K, Anderson J. γδ T cells for cancer immunotherapy: a systematic review of clinical trials. Oncoimmunology. 2014;3:e27572. 24734216 10.4161/onci.27572 PMC3984269

[104] Kouakanou L, Xu Y, Peters C, He J, Wu Y, Yin Z, Vitamin C promotes the proliferation and effector functions of human γδ T cells. Cell Mol Immunol. 2020;17:462–73. 31171862 10.1038/s41423-019-0247-8 PMC7192840

[105] Payne KK, Mine JA, Biswas S, Chaurio RA, Perales-Puchalt A, Anadon CM, BTN3A1 governs antitumor responses by coordinating αβ and γδ T cells. Science. 2020;369:942–9. 32820120 10.1126/science.aay2767 PMC7646930

[106] De Gassart A, Le KS, Brune P, Agaugué S, Sims J, Goubard A, Development of ICT01, a first-in-class, anti-BTN3A antibody for activating Vγ9Vδ2 T cell-mediated antitumor immune response. Sci Transl Med. 2021;13:eabj0835. 34669444 10.1126/scitranslmed.abj0835

[107] Schilbach K, Krickeberg N, Kaiβer C, Mingram S, Kind J, Siegers GM, Suppressive activity of Vδ2^+^ γδ T cells on αβ T cells is licensed by TCR signaling and correlates with signal strength. Cancer Immunol Immunother. 2020;69:593–610. 31982940 10.1007/s00262-019-02469-8 PMC7113223

[108] Traxlmayr MW, Wesch D, Dohnal AM, Funovics P, Fischer MB, Kabelitz D, Immune suppression by gammadelta T-cells as a potential regulatory mechanism after cancer vaccination with IL-12 secreting dendritic cells. J Immunother. 2010;33:40–52. 19952957 10.1097/CJI.0b013e3181b51447

[109] Casetti R, Agrati C, Wallace M, Sacchi A, Martini F, Martino A, Cutting edge: TGF-β1 and IL-15 induce FOXP3^+^ γδ regulatory T cells in the presence of antigen stimulation. J Immunol. 2009;183:3574–7. 19710458 10.4049/jimmunol.0901334

[110] Peters C, Kabelitz D, Wesch D. Regulatory functions of γδ T cells. Cell Mol Life Sci. 2018;75:2125–35. 29520421 10.1007/s00018-018-2788-x PMC11105251

[111] Almeida AR, Correia DV, Fernandes-Platzgummer A, da Silva CL, da Silva MG, Anjos DR, Delta one T cells for immunotherapy of chronic lymphocytic leukemia: clinical-grade expansion/differentiation and preclinical proof of concept. Clin Cancer Res. 2016;22:5795–804. 27307596 10.1158/1078-0432.CCR-16-0597

[112] Di Lorenzo B, Simões AE, Caiado F, Tieppo P, Correia DV, Carvalho T, Broad cytotoxic targeting of acute myeloid leukemia by polyclonal delta one T cells. Cancer Immunol Res. 2019;7:552–8. 30894378 10.1158/2326-6066.CIR-18-0647

[113] Rozenbaum M, Meir A, Aharony Y, Itzhaki O, Schachter J, Bank I, Gamma-delta CAR-T cells show CAR-directed and independent activity against leukemia. Front Immunol. 2020;11:1347. 32714329 10.3389/fimmu.2020.01347 PMC7343910

[114] Fisher J, Sharma R, Don DW, Barisa M, Hurtado MO, Abramowski P, Engineering γδT cells limits tonic signaling associated with chimeric antigen receptors. Sci Signal. 2019;12:eaax1872. 31506382 10.1126/scisignal.aax1872 PMC7055420

[115] Mirzaei HR, Mirzaei H, Lee SY, Hadjati J, Till BG. Prospects for chimeric antigen receptor (CAR) γδ T cells: a potential game changer for adoptive T cell cancer immunotherapy. Cancer Lett. 2016;380:413–23. 27392648 10.1016/j.canlet.2016.07.001 PMC5003697

[116] Oberg HH, Peipp M, Kellner C, Sebens S, Krause S, Petrick D, Novel bispecific antibodies increase γδ T-cell cytotoxicity against pancreatic cancer cells. Cancer Res. 2014;74:1349–60. 24448235 10.1158/0008-5472.CAN-13-0675

[117] Oberg HH, Kellner C, Gonnermann D, Peipp M, Peters C, Sebens S, γδ T cell activation by bispecific antibodies. Cell Immunol. 2015;296:41–9. 25979810 10.1016/j.cellimm.2015.04.009

[118] Ganesan R, Chennupati V, Ramachandran B, Hansen MR, Singh S, Grewal IS. Selective recruitment of γδ T cells by a bispecific antibody for the treatment of acute myeloid leukemia. Leukemia. 2021;35:2274–84. 33526858 10.1038/s41375-021-01122-7 PMC8324575

[119] Mayes PA, Hance KW, Hoos A. The promise and challenges of immune agonist antibody development in cancer. Nat Rev Drug Discov. 2018;17:509–27. 29904196 10.1038/nrd.2018.75

[120] Thommen DS, Schumacher TN. T cell dysfunction in cancer. Cancer Cell. 2018;33:547–62. 29634943 10.1016/j.ccell.2018.03.012 PMC7116508

[121] Ceglia V, Zurawski S, Montes M, Bouteau A, Wang Z, Ellis J, Anti-CD40 antibodies fused to CD40 ligand have superagonist properties. J Immunol. 2021;207:2060–76. 34551965 10.4049/jimmunol.2000704 PMC8490940

[122] Zhang D, Goldberg MV, Chiu ML. Fc engineering approaches to enhance the agonism and effector functions of an anti-OX40 antibody. J Biol Chem. 2016;291:27134–46. 27856634 10.1074/jbc.M116.757773 PMC5207143

[123] Shafique MR, Fisher TL, Evans EE, Leonard JE, Pastore DRE, Mallow CL, A phase Ib/II study of pepinemab in combination with avelumab in advanced non-small cell lung cancer. Clin Cancer Res. 2021;27:3630–40. 33820783 10.1158/1078-0432.CCR-20-4792

[124] Rossi AJ, Khan TM, Hong H, Lesinski GB, Wu C, Hernandez JM. Pepinemab (anti-SEMA4D) in combination with ipilimumab or nivolumab for patients with resectable pancreatic and colorectal cancer. Ann Surg Oncol. 2021;28:4098–9. 33987757 10.1245/s10434-021-10111-0

[125] Brandes M, Willimann K, Moser B. Professional antigen-presentation function by human gammadelta T cells. Science. 2005;309:264–8. 15933162 10.1126/science.1110267

[126] Brandes M, Willimann K, Bioley G, Lévy N, Eberl M, Luo M, Cross-presenting human gammadelta T cells induce robust CD8^+^ alphabeta T cell responses. Proc Natl Acad Sci U S A. 2009;106:2307–12. 19171897 10.1073/pnas.0810059106 PMC2650152

[127] Meuter S, Eberl M, Moser B. Prolonged antigen survival and cytosolic export in cross-presenting human gammadelta T cells. Proc Natl Acad Sci U S A. 2010;107:8730–5. 20413723 10.1073/pnas.1002769107 PMC2889313

[128] Holmen Olofsson G, Idorn M, Carnaz Simões AM, Aehnlich P, Skadborg SK, Noessner E, Vγ9Vδ2 T cells concurrently kill cancer cells and cross-present tumor antigens. Front Immunol. 2021;12:645131. 34149689 10.3389/fimmu.2021.645131 PMC8208807

[129] Larson RC, Maus MV. Recent advances and discoveries in the mechanisms and functions of CAR T cells. Nat Rev Cancer. 2021;21:145–61. 33483715 10.1038/s41568-020-00323-z PMC8353572

[130] Riddell SR, Greenberg PD. The use of anti-CD3 and anti-CD28 monoclonal antibodies to clone and expand human antigen-specific T cells. J Immunol Methods. 1990;128:189–201. 1691237 10.1016/0022-1759(90)90210-m

[131] Testi R, Lanier LL. Functional expression of CD28 on T cell antigen receptor gamma/delta-bearing T lymphocytes. Eur J Immunol. 1989;19:185–8. 2537735 10.1002/eji.1830190129

[132] Ribot JC, Debarros A, Mancio-Silva L, Pamplona A, Silva-Santos B. B7-CD28 costimulatory signals control the survival and proliferation of murine and human γδ T cells via IL-2 production. J Immunol. 2012;189:1202–8. 22732586 10.4049/jimmunol.1200268

[133] Berglund S, Gaballa A, Sawaisorn P, Sundberg B, Uhlin M. Expansion of gammadelta T cells from cord blood: a therapeutical possibility. Stem Cells Int. 2018;2018:8529104. 29707004 10.1155/2018/8529104 PMC5863314

[134] Lafont V, Liautard J, Gross A, Liautard JP, Favero J. Tumor necrosis factor-alpha production is differently regulated in gamma delta and alpha beta human T lymphocytes. J Biol Chem. 2000;275:19282–7. 10764820 10.1074/jbc.M910487199

[135] Phung SK, Miller JS, Felices M. Bi-specific and tri-specific NK cell engagers: the new avenue of targeted NK cell immunotherapy. Mol Diagn Ther. 2021;25:577–92. 34327614 10.1007/s40291-021-00550-6

